# Ultra-Processed Foods in the Food Supply: Prevalence, Nutritional Composition and Use of Voluntary Labelling Schemes

**DOI:** 10.3390/nu17101731

**Published:** 2025-05-20

**Authors:** Edvina Hafner, Maša Hribar, Igor Pravst

**Affiliations:** 1 Institute of Nutrition, Koprska ulica 98, SI-1000 Ljubljana, Slovenia; edvina.hafner@nutris.org (E.H.); masa.hribar@nutris.org (M.H.); 2 Biotechnical Faculty, University of Ljubljana, Jamnikarjeva 101, SI-1000 Ljubljana, Slovenia; 3 VIST–Faculty of Applied Sciences, Gerbičeva cesta 51A, SI-1000 Ljubljana, Slovenia

**Keywords:** ultra-processed foods, food supply, branded foods database, front-of-package labelling, food symbols

## Abstract

Background: Ultra-processed foods (UPFs) represent a substantial part of modern diets, with a growing prevalence in food environments worldwide. Their unfavourable nutritional composition and adverse health effects present growing public health concerns. Methods: This study examines the prevalence of UPFs in the Slovenian food supply, their nutritional quality and the use of different food symbols and labelling schemes on food packaging. A cross-sectional analysis was conducted using the representative Slovenian branded foods database. A total of 23,173 prepacked foods and beverages were categorised into levels of processing according to the NOVA classification system. The nutritional composition of UPFs was compared to less processed products within 16 narrow subcategories. Additionally, the prevalence in the use of front-of-package nutrition labelling (FOPNL) and subjectively nutrition-related elements (SNREs) (such as EU Organic, Vegan labels etc.) were assessed across different food categories and processing levels. Results: Results show that UPFs represent 54.5% of the available products in the Slovenian food supply, with the highest prevalence in Confectionery (93%), Bread and bakery products (83%), Meat, meat products and alternatives (77%) and Convenience foods (74%). Comparison of nutritional composition indicated that UPFs had significantly poorer nutritional composition compared to less processed counterparts, including higher levels of sugar, salt and saturated fats, and a lower protein content. Breakfast cereals, Snack foods, Meat alternatives and Pre-prepared salads and sandwiches showed the most significant differences between UPFs and less processed counterparts. Analysis of the prevalence of symbols and labelling schemes revealed that 33.8% of products carried at least one FOPNL (15.0%) or SNRE (19.1%), with SNREs being more prevalent on less processed products and FOPNL predominantly used on UPFs (*p* < 0.05). The most prevalent SNRE was the EU Organic logo (12.7%), followed by the Vegan (4.7%) and Non-GMO (3.1%) logos, whereas the most frequent FOPNL was Reference Intakes (RI), presenting only energy value RI-Energy (12.5%), followed by nutrient-specific RI (1.6%), while other FOPNL were scarce and limited to certain categories. An additional comparison of visual presentation highlighted the potentially selective use of voluntary FOPNL to improve product framing. This raises concerns about their role in guiding consumer choices versus serving as marketing tools, especially when it comes to UPFs. Conclusions: Our findings highlight the need for monitoring UPFs in the food supply together with harmonised, mandatory labelling regulations to ensure transparency and empower consumers to make healthier choices.

## 1. Introduction

Ultra-processed foods (UPFs) present a considerable part of the modern diet, accounting for 14–44% of daily energy intake in Europe and over 50% in the United States [1,2,3], and their variety and availability continue to rise [4]. The growing share of UPFs in the food supply is also reflected in daily diets, contributing to their high proportion of total food intake in many countries [5]. Understanding the availability of UPFs in the food supply is crucial for assessing their impact on public health and diet quality [6,7]. This is particularly noteworthy since UPFs are linked to non-communicable diseases (NCDs), such as type 2 diabetes and cardiometabolic diseases [8]. UPFs are commonly characterised by unfavourable nutritional composition, with higher content of energy and critical ingredients, such as sugar, salt and saturated fats [9]. Knowing the nutritional composition of the available UPFs helps identify the major health risks and food reformulation opportunities [10]. Even though research on UPFs’ effect on health is still limited and their definition remains debated [11], the NOVA categorization is the most recognised and scientifically supported tool to define UPFs [12]. The NOVA classification system categorises foods into four groups based on the extent of processing, ranging from minimally processed foods (NOVA 1) to ultra-processed foods (NOVA 4). NOVA 4 includes products with ingredients typically used in the industry, such as sweeteners, emulsifiers, flavourings and certain additives. Based on NOVA, many dietary guidelines already recommend limiting consumption of UPFs in order to maintain a healthy and sustainable diet [13].

UPFs are commonly sold in convenient packaging, and equipped with highly attractive food labelling that is intentionally crafted to encourage purchasing decisions and impact food choices [14]. Food labelling is therefore a noteworthy factor in shaping consumers’ diets and consumption of UPFs [15,16]. Food labelling connects consumers and producers, aiming to support informed food choices [17]. It is processed through a dual-processing approach: a fast, intuitive heuristic system and a slower, analytical system [18,19]. A modern, fast-paced lifestyle and limited nutritional knowledge lead consumers to rely on heuristics when making dietary choices, often evoked by graphic elements on food labels [20]. This has resulted in the rise of front-of-package nutrition labelling (FOPNL) and other symbols and labelling schemes over the past two decades [21]. While the industry has long used heuristics for marketing, policymakers have increasingly embraced this approach as a tool to encourage healthier choices in the past decade [22].

Symbols and labelling schemes that visually sum up information about the product can be divided into two groups [21]. First are objectively nutrition-related elements including FOPNL, which aim to summarise nutritional composition information for easier and quicker comprehension, ultimately promoting healthier food choices [23]. FOPNL schemes can notably vary between interpretative or non-interpretative. Interpretative FOPNL like the Multiple Traffic Light System (MTL) [24], Nutri-Score [25], Protect Health [26] and Keyhole [27] use colours and logos to engage a heuristic approach [28]. Non-interpretative labels, such as Reference Intakes (RI) [29], provide nutritional summaries but require more knowledge and time to interpret, evoking the analytical system. Schemes can also differ in presenting values per 100 g or per portion. As a result of these differences, various stakeholders advocate for different models [30]. Therefore, despite the efforts engaging the European Commission to introduce unified FOPNL in the European Union (EU), the use of FOPNL remains unharmonised and voluntary, with a variety of national and commercial schemes [31], and no system to monitor their use in real life.

A similar situation is seen in the second group, which includes subjectively nutrition-related elements (SNREs) that provide information to consumers that is not directly related to nutritional composition, but expose other attributes of food products [21], such as logos for organic production, origin, vegan, traditional specialties etc. Most of such labelling schemes are also voluntary and unharmonised. Exceptions include the EU Organic logo [32] and EU quality schemes such as geographical indicators and traditional specialities [33].

The increased quantity of abovementioned labelling schemes is raising a question about whether they truly help consumers in making informed food choices or whether they are potentially misused for marketing [20]. Voluntary practices, as seen in food reformulation, may not always deliver expected results [34]. This can be particularly problematic if labels promote foods that are in conflict with dietary guidelines, such as UPFs [35]. A study from Brazil showed that a high prevalence of health-focused marketing techniques on UPFs is alarming [36]. Monitoring voluntary labelling practices is essential to ensure that they are being used effectively to guide consumers towards healthier choices, rather than being potentially exploited for marketing of less favourable products, such as UPFs.

The first objective of our study was therefore to assess the prevalence of UPFs in the Slovenian food supply and their nutritional composition in comparison to less processed products. The second objective was to investigate the prevalence and differences in the use of the food symbols and labelling schemes such as FOPNL and SNRE between food categories, and in connection with processing levels. To identify UPFs in our data, we used the NOVA system as the most relevant food processing classification in the literature.

## 2. Materials and Methods

### 2.1. Dataset and Food Categorisation

This study used a large, branded food database to analyse the prevalence of UPFs, their nutritional composition and prevalence of different food symbols and labelling schemes. We used a representative Slovenian branded foods database, collected within the CLAS (Composition and Labelling Information System), which contains data on prepacked foods and beverages from major retailers in Slovenia. Detailed methodology on data collection and processing in the CLAS is described elsewhere [10]. In brief, photographs of food labels are collected in food stores using a specialised mobile app and transferred to the CLAS cloud for data extraction. Data in CLAS represent cross-sectional data on available prepacked foods from all major retailers with a nation-wide network of shops. This study used the full 2020 CLAS dataset, which covered six major food retailers in Slovenia. According to the aim of this study, we used available data from food labelling on nutritional composition, ingredient list and presence of different labelling schemes. Products were categorised into 14 main food categories and 69 subcategories according to the food categorisation system proposed by the international Global Food Monitoring Group [37].

Our initial database included 28,028 prepacked food products and drinks. Of those, 23,173 had sufficient data to determine the level of processing and were therefore included in our study.

### 2.2. Classificaton of UPFs and Analysis of Nutritional Composition

For the classification of foods based on the level of processing, we used the NOVA system [12]. Each product was classified into one of the NOVA groups, based on their ingredient list. NOVA classifies food products into four groups from less processed foods (NOVA 1–3) to UPFs (NOVA 4):

- 1: un-processed and minimally processed foods: foods that are in their original form or are only dried, frozen, crushed, pasteurised etc., with no addition of other ingredients.
- 2: processed culinary ingredients: foods that are rarely used on their own, mainly used for preparation, cooking and seasoning of foods in group 1: sugar, salt, oils and fats.
- 3: processed foods: products that are a combination of foods from groups 1 and 2: e.g., cheeses, breads, canned vegetables.
- 4: ultra-processed foods (UPFs): foods that have formulations that are typically used only in the food industry and are mostly made from substances extracted from foods: e.g., varieties of sugars (invert sugar, fructose, dextrose), hydrogenated oils, hydrolysed proteins, sensory additives (flavours, colours, emulsifiers, sweeteners). These commonly include products such as confectionery, prepacked meals, crisps, baked products etc.

Differences in nutritional composition between less processed products and UPFs were assessed by comparing energy value, content of sugar, salt, fat, saturated fats, proteins and overall nutritional profile. The overall nutritional profile was assessed using the updated 2023 version of Nutri-Score (NS2023) [38,39]. NS2023 evaluates foods based on negative (energy, sugars, saturated fats, salt) and positive attributes (dietary fibre, protein, % fruit, vegetables, legumes). The final score determines a grade from A (most healthy) to E (least healthy). The detailed methodology on nutrient profiling of foods in the CLAS dataset is descried elsewhere [40].

### 2.3. Use of Symbols and Labelling Schemes on Food Packaging

**Figure 1:**
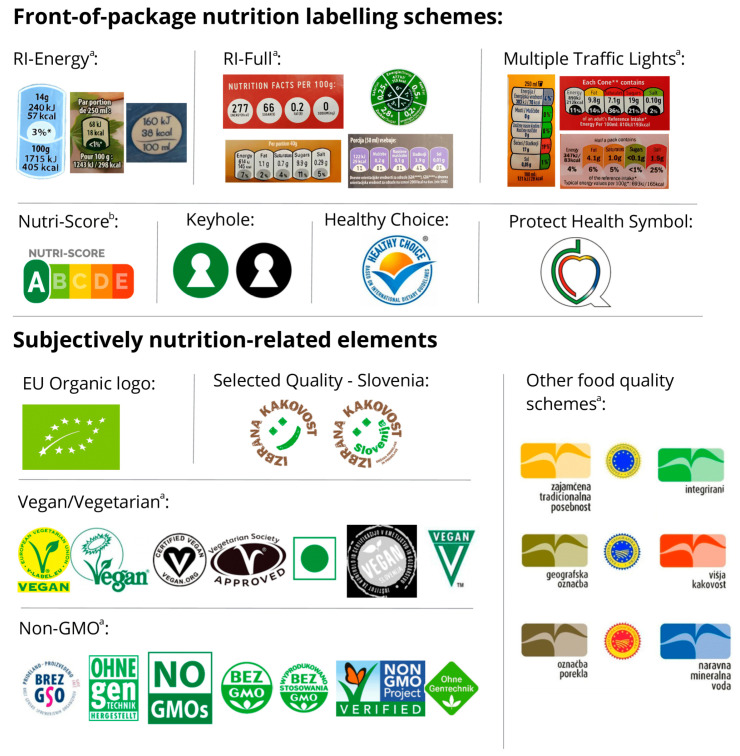
Front-of-package labels and subjectively nutrition-related elements included in the food labelling review of prepacked foods and beverages from the Slovenian food supply (*n* = 23,173). (RI-Energy: Reference Intakes displaying only energy value; RI-Full: nutrient-specific Reference Intakes; Non-GMO: non-genetically modified organisms; ^a^ All different labelling schemes were treated together; ^b^ Including all variations of Nutri-Score (from A-most healthy to E-least healthy)).

Each product label underwent examination for the presence of food symbols and labelling schemes (Figure 1). These included FOPNL, which represents nutritional food qualities, and SNREs that represent other “non-nutritional” food qualities. The FOPNL that were included in our study were Reference Intakes (different variations including version presenting only energy value (RI-Energy) or nutrient-specific version (RI-Full)), Multiple Traffic Light System (MTL), Nutri-Score (NS), Keyhole (KH), Healthy Choice (HC) and the Slovenian Protect Health (PH) symbol. The SNREs included in this study were the EU Organic logo, variations of Vegan and Vegetarian label, non-genetically modified organisms (Non-GMO), the Selected Quality—Slovenia (SQ) label and other food quality schemes. We only included labelling schemes that have a defined symbol certification process. The full list of labelling schemes and their variations is presented in Figure 1.

### 2.4. Data Processing and Analyses

Data were processed using Microsoft SQL Server Management Studio 13.0, Microsoft Excel 2019 (Microsoft, Redmond, Washington, DC, USA) and the Composition and Labelling Information System—CLAS (Institute of Nutrition, Ljubljana, Slovenia). Statistical analyses were performed with R: A Language and Environment for Statistical Computing 4.4.2 (R Core Team) and Microsoft Excel 2019 (Microsoft).

First, we assessed the distribution of NOVA across branded foods (*n* = 23,173). Then, we analysed the differences in nutritional composition between less processed products (NOVA 1–3) and UPFs (NOVA 4). While the categorization of UPFs according to NOVA is often category specific [12], analysing their nutritional composition compared to less processed products can be challenging. Our previous research on the nutritional composition of foods in the Slovenian food supply had shown that food categories commonly associated with less processed products typically have a better nutritional composition than those with more processed products (for example plain yoghurt vs. flavoured yoghurt) [41]. However, that study did not apply a formal processing classification. In the present study, we systematically used the NOVA classification to compare UPFs and less processed products within specific, nutritionally relevant food subcategories, with similar use products. We focused on subcategories with less than 75% of either UPFs or less processed products that were large enough for a meaningful comparison (*n* > 30). This enabled us to determine if, among similarly used products, a lower processing level is related to better nutritional quality. The subsample for this part of the study consisted of 4860 products from 16 food subcategories. Differences in the nutritional composition between UPFs and less processed products within these (sub)categories were analysed using the nonparametric Mann–Whitney U test (*p* < 0.05) for energy value, sugar, salt, fats, saturated fats, proteins and NS2023 profiling score.

In the second part of this study, we assessed the distribution of different food symbols and labelling schemes across prepacked products and their connection to the processing level. A two-tailed z-test (*p* < 0.05) was used to compare the prevalence of FOPNL on UPFs and less processed products. Differences in SNREs and their connection to UPFs were analysed descriptively rather than statistically. This approach was used because, while all variations of FOPNL refer to nutritional composition of the product and can be put on most products, SNREs are much more diverse, addressing very different aspects of food qualities or properties. Furthermore, some symbols, such as vegan labels, cannot be displayed on a variety of products like dairy and meat, limiting their applicability.

## 3. Results

### 3.1. Prevalenece of UPFs and Their Nutritional Composition

**Figure 2:**
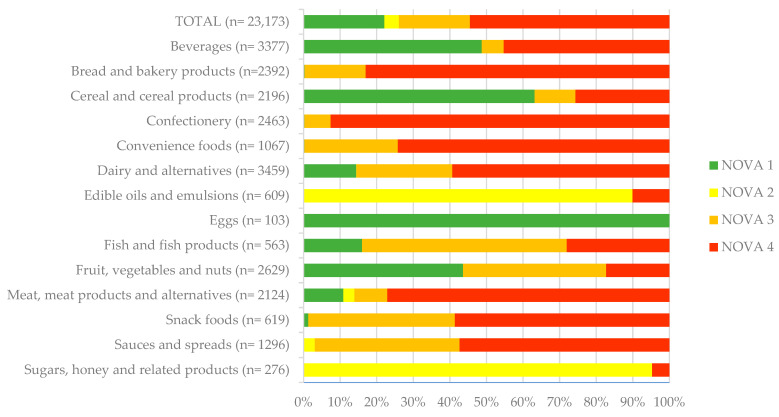
Distribution of NOVA processing levels across main food categories in the 2020 Slovenian food supply (*n* = 23,173). Exact values and subcategory results are provided in Supplementary Material Table S1.

Based on the NOVA classification, our sample consisted of 54.5% UPFs (*n* = 12,630) (NOVA 4) products and 45.5% less processed products (*n* = 10,543), of which 22.1% (*n* = 5130) were NOVA 1, 3.9% (*n* = 912) NOVA 2 and 19.4% (*n* = 4501) NOVA 3 (Figure 2). The main categories with the highest prevalence of UPFs were Confectionery (93%), Bread and bakery products (83%), Meat and meat products (77%) and Convenience foods (74%). Categories with the lowest prevalence of UPFs were Fruit, vegetables and nuts (17%), Edible oils and emulsions (10%), Sugar, honey and related products (5%) and Eggs (0%).

Among 69 food subcategories, 23 had more than 75% UPFs among their offerings, while 28 had over 75% less processed foods (NOVA 1–3). This shows that UPFs as defined by NOVA are often category specific. Categories like Pizza, Soft drinks and Biscuits are dominated by UPFs (100%, 98% and 92%, respectively), while the contrary applies for categories like Fresh fruit and vegetables (0%). However, in certain categories (*n* = 18), consumers have more ability to choose between UPFs and less processed options. Among these subcategories are Cheese and processed cheese (28% UPFs), Coffee products (32% UPFs), Sauces (55% UPFs), Crisps and snacks (59% UPFs) and Breakfast cereals (70% UPFs). The full distribution of NOVA processing levels by food categories and subcategories is available in Supplementary Material Table S1.

For the analysis of nutritional composition (Table 1) in relation to the level of processing, we analysed the abovementioned 18 food subcategories with less than 75% of either UPFs or less processed products, where consumers have meaningful options for choosing either less or more processed foods. Two categories were excluded because of the lack of the nutritional composition information (Coffee and Syrups). In the end, we created a subsample (*n* = 4860) from 16 food subcategories with sufficient data and enough (*n* > 30) of both UPFs and less processed products.

Contrary to previous studies based on general perceptions, we employed the NOVA classification to ascertain whether, in some subgroups of similarly utilised foods, UPFs differ nutritionally from less processed alternatives. The study results indicate that in selected food categories, UPFs generally had poorer nutritional composition compared to less processed alternatives. In 12 out of 16 categories, less processed foods had a significantly better nutritional profile based on the NS2023 profiling score (*p* < 0.05). Exceptions included Cream, Meat alternatives and Spreads, where the difference did not reach statistical significance. Processed fish products were the only category where UPFs had a better NS2023 profiling score. This result likely reflects the slightly higher variety of products within the category, as more processed items (such as canned fish salads with added vegetables) may receive a better NS2023 score than less processed fish in oil or brine (higher fat and salt).

UPFs had a higher content of sugar across 13 categories, salt in 9 categories, saturated fats in 6 categories and energy value in 6 categories. Fat content was significantly higher in UPFs only in Breakfast cereals. In 8 categories, UPFs also had a lower protein content, compared to less processed foods.

Breakfast cereals showed the most pronounced differences, with UPFs having significantly higher energy, fat, saturated fats, sugar and salt; a lower protein content; and an overall worse nutritional profile according to the NS2023 profiling score. Similar trends were observed in the categories of Snack foods, Meat alternatives, and Pre-prepared sandwiches and salads for most of the analysed characteristics. Considerable differences were also observed in Cheese and processed cheese, where less processed items had a higher energy value, fat and saturated fats content but also a higher protein content and less sugar and salt compared to UPFs. Similarly, less processed Milk and milk drinks had higher fat and saturated fats but lower sugar, salt, and protein, and an overall better NS2023 score. Milk alternatives were the only category where the sugar content was significantly higher in less processed products than in UPFs. They also had a worse NS2023 score, but the difference was not significant.

### 3.2. Prevalence of Graphical Elements and Their Connection to UPFs and Nutritional Quality

Our examination of food labels across the entire sample (*n* = 23,173) identified 9080 food symbols and labelling schemes that were present on 33.8% of the investigated prepacked foods and drinks. FOPNL were found on 15.0% *(n* = 3494) of products, while SNREs were found on 19.1% (*n* = 5586) of products. Table 2 displays the prevalence of FOPNL and SNREs across different food categories and levels of processing.

In general, SNRE symbols were more frequently used than FOPNL and more common on less processed products. The highest penetration was observed for the EU Organic logo (12.7%), followed by Vegan (4.7%), Non-GMO (3.1%), SQ (2.6%) and other food quality schemes (0.9%). The highest prevalence of SNRE symbols was found in Edible oils and emulsions (32.3%), Dairy (31.2%), Eggs (27.2%) and Cereals and cereal products (24.1%). The EU Organic logo appeared in most categories but was particularly common in Cereal flakes and bran (67%), Dairy and meat alternatives (32–64%), Fresh fruit (35%), Spreads (39%), Nut spreads (38%) and Syrups (41%). Some symbols were predominantly found in specific food categories. Non-GMO was most prevalent in Eggs and Dairy (14% and 9%, respectively). Other food quality schemes were mainly used on Meat, meat products and alternatives (*n* = 74), Edible oils and emulsions (*n* = 37) and Dairy and alternatives (*n* = 34). The SQ label was most frequently displayed on Meat, meat products and alternatives (*n* = 109) and Dairy and alternatives (*n* = 483).

When assessing foods displaying FOPNL, we found they were more commonly displayed on UPFs (19.6%) compared to less processed products (9.4%) (*p* < 0.05). This significant trend was observed in 8 of the 14 main food categories, including Beverages, Cereals and cereal products, Confectionery, Convenience foods, Dairy and alternatives, Meat and alternatives, Snack foods and Sauces and spreads. An exception was found in the Fruit and Vegetables category, where less processed products more frequently displayed FOPNL (8.9%) compared to UPFs (5.5%), primarily due to the labelling of frozen vegetables. Categories with the highest prevalence of FOPNL were Convenience foods (25.1%), Snack foods (31.2%), Bread and bakery products (20.6%) and Dairy (19.9%). At the subcategory level, some categories with higher prevalence of displaying FOPNL include Breakfast cereals (41.9%), Flavoured yoghurts (25.8%), Ice creams and edible ices (33.0%) and Crispy bread (30.2%). Regarding specific FOPNL, RI Energy was the most used (12.5%), followed by RI Full (1.6%). Other FOPNL appeared less frequently and appeared on a smaller number of products, often limited to specific categories. For example, MTL (*n* = 82) was mostly found on Soft drinks, NS (*n* = 68) on Flavoured yogurts and Protect Health (*n* = 72) on Plain and Flavoured yogurts and Cooking oils.

### 3.3. Case Study: Comparison of Visual Presentation of Various Voluntary FOPNL for Selected Foood Products

**Figure 3:**
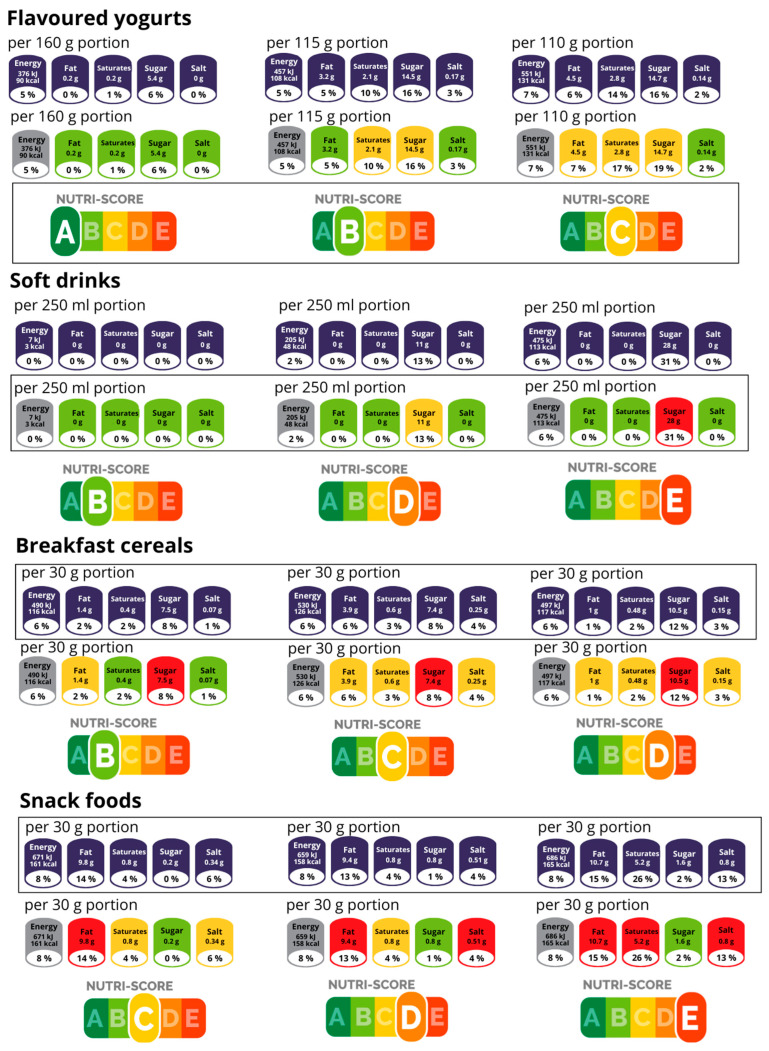
Visual representation of front-of-package nutrition labelling application across Soft drinks, Yogurts, Breakfast cereals, and Snack foods to assess perception optimization. Rectangles highlight the FOPNL typically used on the product category, while the other two represent alternatives that could have been applied. Letters from A to E represent five Nutri-Score grades.

Although our data showed a lower prevalence of FOPNL use, certain trends in their application can be observed. A selection of specific FOPNL might be a strategic marketing decision of a manufacturer, affecting how a specific product is presented to consumers. We conducted a case study where three different FOPNL that were mostly found in some categories were also applied to product categories, where such an FOPNL is not common. RI-Full (mostly seen on Breakfast cereals and Snack foods), NS (mostly used on Flavoured yogurts) and MTL (mainly used on Soft drinks) were applied across all four food categories. For this assessment, we selected three products with varying nutritional composition from each category that originally carried a specific FOPNL and generated visual presentation of the remaining two FOPNL. As presented in Figure 3, the use of a certain FOPNL can frame a product very differently, presenting an opportunity for manufacturers to make it more attractive to consumers. Yogurts appeared best under NS (according to the model, which was applicable at the time of data collection), as the mostly aggregated A and B ratings provided an overall positive impression, minimizing focus on nutrients of concern within this category. Soft drinks were more favourably presented under the MTL system, as three green indicators could create a health halo effect [42], diverting attention from the red sugar warning, which is the key critical ingredient in this category. Meanwhile, Breakfast cereals and Snack foods more often used RI-Full, where small portion sizes enable presentation of lower content numbers for nutrients of concern (fats, sugar, salt), whereas MTL or NS labelling would show a less favourable grade.

## 4. Discussion

The study results revealed that over half of prepacked products in the Slovenian food supply are UPFs. This study also showed that similar products typically had better nutritional composition when they were less processed. Additionally, one third of the products featured at least one food symbol or labelling scheme on the packaging, with SNREs (particularly the EU Organic logo) being more common than FOPNL, and mostly found on less processed products. In contrast, FOPNL were scarce (especially interpretative types) and appeared more often on UPFs (*p* < 0.05). A comparative case study with different FOPNL highlighted a potential knowledge gap in the selective use of voluntary FOPNL, highlighting the need for further examination and monitoring.

The prevalence of the UPFs in the Slovenian food supply is similar to findings from Chile (54%) [43], but lower than the prevalence found in Canada (74%) [44], the United States (US) (71%) [45], South Africa (76%) [46] and Greece (70%) [47]. Due to differences in sampling methods and acknowledged limitations of the NOVA classification [47], which might not offer sufficient ability to discriminate between products, cross-country comparisons should be interpreted with caution. Availability of UPFs in the food supply has been increasing over time [48], and food environment plays an important role in shaping consumers’ diets [49]. For instance, in the United States, the share of the UPFs in the diet has risen from 53.5% to 57% in the last 15 years. This reflects a generation shift and the past growth of the UPF industry, which exposed today’s adults to UPFs during childhood, shaping their long-term eating habits [3]. Recent estimates suggest that about 23% of the Slovenian energy intake comes from UPFs, a figure expected to rise, following European trends (up to 44%) [2]. This is concerning as UPFs are often associated with poor nutritional composition and risk of a variety of NCDs [8,9], which are highly prevalent in Slovenia and many other countries [50].

UPFs are commonly characterised by an unfavourable nutritional composition, and particularly concentrated in specific food categories. Our study found that UPFs are most prevalent in food categories that dietary guidelines recommend limiting [51]. This includes products like soft drinks, biscuits, processed meats, chocolate, desserts, and ice cream, where the proportion of UPFs exceeded 90%. However, some of the categories include products with similar use but of different levels of processing, leaving consumers with an actual choice between UPFs and less processed products. One such category is breakfast cereals, a common household staple with a wide variety available on the market [52]. In such selected food categories, where there is a presence of both UPFs and less processed foods, we found that UPFs tended to have a poorer nutritional profile. This is particularly noteworthy because UPFs tend to be very convenient and cheaper [53], making them very attractive for consumers. Consequently, even in categories with more diverse choices, the poor composition of UPFs may disproportionately impact vulnerable population groups, who struggle with NCDs even more [54,55]. On the other hand, we have to emphasise that not all UPFs have unfavourable nutritional composition, and recent studies showed that some UPFs (such as soft drinks and processed meats) might have a worse effect on health than UPFs from other categories (such as bakery products and dairy). Moreover, the relative contribution of such products to the national food basket is an important consideration in assessing UPF public health relevance [56,57]. Monitoring the prevalence and nutritional composition of UPFs, together with their relevance in national diets, is therefore also important in the management of NCDs [4].

In the second part of this study, we examined the use of food symbols and labelling schemes across categories and processing levels. SNREs were more common on less processed products, with the EU Organic logo being the most dominant (12.7%). This was one of few fully regulated EU-harmonised logos in our study [32]. A higher prevalence of this logo on less processed products can be explained, as such products contain fewer ingredients that need to be organic, unlike multi-ingredient UPFs, where certifying numerous components may be more challenging [58]. Despite its widespread use, studies suggest that only 56% of EU consumers recognise the Organic logo, raising concerns about labelling effectiveness [59]. If a well-established, regulated label lacks consumer awareness, the situation may be even more complex for other, less standardised labels. One of such are vegan/vegetarian logos, which were found on 4.7% of products from our database. The plant-based sector is on the rise, increasing the demand for vegan-labelled products [60]. However, in contrast to the EU Organic logo, vegan and vegetarian claims are not yet regulated in the EU [61]. Our study identified many different labelling schemes communicating similar information, highlighting the inconsistency in how these products are labelled. Given the potential for a health halo effect [62], clearer regulatory guidance may become necessary as we anticipate that the growth of such products will continue to increase. Non-GMO labelling follows a similar pattern. While EU regulations on GMOs are already quite strict (and most foods in the EU do not contain GMOs) [63], Non-GMO labels have become more common despite lacking regulation [64]. In over 50% of cases, the Non-GMO logo appeared alongside the EU Organic logo, which by its strict regulations already prohibits the use of GMOs [58]. This suggests that such logos may be used to further highlight this characteristic to consumers, perceiving it as safer, as seen in the United States [65]. Other food quality schemes and SQ were less common, mainly found on dairy, meat and cooking oils, reflecting sector-specific participation in these schemes. In Slovenia, the SQ scheme was initially introduced to dairy and meat products in 2016, and further expanded to the fruit and grain sector in 2019 and 2023, respectively [66], meaning that its usage probably increased and suggesting closer monitoring in the future.

For the FOPNL, we found that they were more prevalent on UPFs than on less processed products. Similar findings in Canada, before mandatory FOPNL implementation, raised concerns about the effectiveness of voluntary labelling in supporting public health [67]. Interestingly, study results showed that consumers are mostly exposed to FOPNL through food categories that have a high percentage of UPFs and are typically considered less healthy. This includes products like soft drinks, biscuits, chocolate, sweets and flavoured yogurts. A study in Serbia reported similar results; they found notable differences in the use of FOPNL, with UPF-associated categories (e.g., biscuits, soft drinks) using them more frequently than less processed ones (e.g., milk, cheese) [68].

Several factors may influence the degree to which FOPNL are implemented and which types are more commonly used. A higher prevalence of FOPNL in the category of beverages might be impacted by actions of the Slovenian Chamber of Commerce, which published a responsibility pledge for the sector of beverages manufacturers in 2015, which engages its members to display FOPNL [69]. We found that products labelled with FOPNL mostly used RI-Energy (followed by RI-Full on a much smaller scale), while all other types of FOPNL were scarce. This reflects the voluntary nature of FOPNL implementation, as the industry has consistently shown greater support to non-interpretative labels [70]. Studies shows that products with interpretative FOPNL were normally healthier than those displaying non-interpretative and that implementation of non-interpretative labels can be twice as fast as interpretative [71]. Since non-interpretative FOPNL have a limited effect on consumer purchase decisions, such labels do not engage manufacturers to reformulate and may motivate them to use it on less healthy products [70].

Other FOPNL were rarely used and mostly limited to certain categories. Our case-based analysis explored how certain FOPNL formats are applied in selected categories and how this may relate to product framing rather than objective nutritional quality. The categories were chosen based on the frequent presence of a specific FOPNL. Soft drinks appeared more favourable under the frequently used MTL due to multiple green lights. The NS (2020 version) was most applied to flavoured yogurts, predominantly with ratings A and B. RI-Full was most frequently used on snack foods and breakfast cereals with small portion sizes. These labelling options can be understood through well-established cognitive biases and behavioural theories. Since food labels are increasingly processed using heuristics, the likelihood of cognitive biases becomes more pronounced [72]. Framing theory shows that the presentation of information on food labels shapes consumer interpretation [73]. Framing products with a green colour using MTL and NS may create a health halo [42], while low portion sizes when using RI-Full might create an anchor that underestimates actual intake [28]. This may act as a nudge to subtly influence purchasing decisions [20]. Such a psychological frame can also be supported with previous studies, suggesting that some labelling practices can be used as a marketing strategy by the food industry to present food products as healthier [74]. While this analysis is illustrative and limited in scope, it raises important questions about the strategic use of FOPNL and the implications for consumers seeking healthier choices.

The inconsistent and selective use of voluntary labelling schemes raises concerns about their effectiveness in promoting informed consumer choices. The variation in FOPNL applications across product categories and their more prevalent use on UPFs suggests that labels may fail to support informed consumer choices and could even be misleading. This raises concerns about their overall effectiveness as a public health tool. To address these limitations, we emphasise the need for a harmonised and mandatory FOPNL system across Europe that ensures consistent application across all product categories. Evidence from Chile shows that the implementation of mandatory FOPNL has led to increased consumer awareness, increased product reformulation and decreased sales in unhealthy foods [75,76,77]. These outcomes highlight the potential benefits of a regulated approach, especially when combined with public education campaigns aimed at strengthening consumer understanding and promoting healthier dietary choices.

The main advantage of our study is a very large and representative food composition and labelling dataset, along with a comprehensive approach to analysing UPFs, including product offering, nutritional composition and labelling practices. We analysed the use of a wide variety of symbols and labelling schemes, for which the data on their market implementation is lacking. Such insights can highlight gaps in voluntary labelling practices and identify new areas for research. Some limitations should also be noted. The used NOVA classification to identify UPFs faces criticism that it lacks the ability in differentiating processing levels and may have inconsistency across different raters [11]. To minimise subjectivity, we applied an ingredient-based approach. While NOVA might not be ideal, it remains the most widely used and studied classification system, and is also included in some dietary guidelines [13]. Regarding the selection of investigated symbols and labelling schemes, we should note that a variety of schemes that address other qualities of food were not included in this study, such as green labels (Fair Trade, Rainforest Alliance, Carbon label etc.) and other dietary indicators like gluten free, lactose free etc. We should also mention that the proportion of UPFs, and prevalence of the use of FOPNL on food packaging, were only assessed for prepacked foods available in food markets, and without correction for different market-shares; we did not investigate non-prepacked foods or prepacked foods that are distributed in other environments. Lastly, while we observed some trends in voluntary FOPNL adoption, the sample size of products labelled with certain FOPNL was relatively small, warranting further investigation before drawing conclusions. In addition, the case study analysis was limited to a few selected categories, chosen based on the prevalence of specific FOPNL formats, which is not representative of broader labelling practices across the food supply. The observational nature of this case should be interpreted as illustrative rather than generalizable. Further research using systematic sampling and larger datasets is needed to assess whether the observed patterns reflect broader marketing strategies or consistent framing effects across categories.

## 5. Conclusions

We can conclude that the prevalence of UPFs in the Slovenian food supply is high and comparable to findings from other countries, and that the nutritional composition of UPFs within selected categories is generally poorer than their less processed counterparts. The implementation of different voluntary symbols and labelling schemes on food packages varies across different food categories and processing levels and should be further monitored and regulated. A major challenge in this area is the voluntary use and lack of harmonization of such labelling schemes. To avoid misleading practices and to guide the harmonisation of food labelling, further research is needed to investigate how consumer perceptions of a food product vary based on different FOPNL formats.

## Figures and Tables

**Table 1:** Comparison of median energy value, nutrient content and Nutri-Score 2023 profiling score between ultra-processed (NOVA 4) and less processed (NOVA 1–3) foods in selected food categories (*n* = 4860).

Category	NOVA 4/NOVA 1–3							
	N	Energy (kJ/100 g)	Fat (g/100 g)	Saturates (g/100 g)	Sugar (g/100 g)	Salt (g/100 g)	Proteins (g/100 g)	Nutri-Score 2023 (Profiling Score)
Breakfast cereals	338/148	1667/1596 ^c^	8.4/5.5 ^b^	2.5/1.3 ^c^	20.0/13.0 ^c^	0.38/0.16 ^c^	8.9/9.9 ^c^	11/5 ^c^
Canned fruit	34/77	2890/277	0.1/0.1	0/0	15.0/13.8 ^a^	0.02/0.01	0.4/0.5 ^a^	3/2 ^b^
Cheese and processed cheese	229/602	1090/1387 ^c^	23.0/26.0 ^c^	15.0/17.3 ^c^	3.0/0.5 ^c^	1.80/1.50 ^c^	14.0/23.0 ^c^	16/14 ^c^
Cream	72/82	1169/896	26.0/21.0	17.0/14.8	3.5/3.3 ^b^	0.10/0.10	2.5/3.0 ^c^	15/14
Crispy bread	67/147	1769/1744	8.9/10.0	2.4/1.3 ^b^	2.6/2.3	1.80/1.60	11.4/11.1	15/12 ^b^
Fresh filled pasta	102/38	946/888	5.0/5.4	1.9/2.6	2.0/1.1 ^a^	1.10/0.97 ^b^	8.0/8.2	7/5 ^a^
Meat alternatives	104/58	961/749 ^c^	13.0/8.4 ^c^	1.7/1.5 ^a^	1.0/0.4 ^c^	1.60/0.44 ^c^	15.2/13.9	10/−1 ^c^
Milk alternatives	86/93	1889/220 ^a^	1.7/1.4	0.3/0.2 ^c^	2.9/4.8 ^a^	0.10/0.10	0.6/0.7	4/8
Milk and milk drinks	77/162	268/264 ^c^	1.1/3.5 ^c^	0.8/2.0 ^c^	9.3/4.7 ^c^	0.14/0.10 ^c^	3.6/3.3 ^c^	7/2 ^c^
Pre-prepared salads and sandwiches	96/32	951/761 ^c^	12.0/11.0	2.2/1.5 ^c^	2.3/1.8 ^a^	1.35/1.05 ^a^	8.5/8.4	7/4 ^c^
Processed fish products	74/69	858/756	9.5/12.0	1.7/2.0 ^a^	1.5/0.5 ^c^	1.24/2.00	11.0/15.0 ^c^	7/17 ^c^
Ready meals	215/86	624/690	6.7/5.6	1.7/1.0 ^a^	1.4/1.4	1.10/0.85 ^c^	7.0/7.0	4/3 ^c^
Sauces	431/354	515/424 ^c^	3.1/4.2	0.3/0.4	7.0/4.3 ^c^	1.75/1.60	1.5/2.3 ^c^	13/9 ^c^
Side dishes	119/85	774/696	7.2/4.9	1.4/1.1	4.4/0.8 ^c^	0.90/0.70 ^a^	5.2/3.7	6/4 ^b^
Snack foods	363/255	2097/2029 ^a^	26.7/24.0	3.2/3.0	2.9/0.9 ^c^	1.90/1.50 ^c^	6.7/7.3 ^a^	17/14 ^c^
Spreads	79/86	925/1071 ^c^	20.0/20.5	2.8/2.7	1.7/1.2 ^b^	1.20/1.17	4.5/7.0 ^c^	8/6

Nonparametric Mann–Whitney U test was used to compare differences between UPFs (NOVA 4) and less processed products (NOVA 1–3): ^a^ (*p* < 0.05); ^b^ (*p* < 0.01); ^c^ (*p* < 0.001).

**Table 2:** Prevalence of front-of-package nutrition labelling (FOPNL) and subjective nutrition-related elements (SNREs) across main food categories and processing levels in the investigated branded foods dataset (Slovenia, *n* = 23,173). Subcategory results are provided in Supplementary Material Table S2.

	FOPNL (*n* (%)) ‡						SNRE (*n* (%))				
	Any FOPNL	RI-Energy	RI-Full	PH	NS	MTL	Organic	Vegan	Non-GMO	Other	SQ
Total * (*n* = 23,173)	3474 (15.0)	2887 (12.5)	379 (1.6)	72 (0.3)	68 (0.3)	82 (0.4)	2950 (12.7)	1099 (4.7)	723 (3.1)	200 (0.9)	614 (2.6)
NOVA 1–3 (*n* = 10,543)	994 (9.4)	782 (7.4)	159 (1.5)	47 (0.4)	6 (0.1)	9 (0.1)	2265 (21.5)	538 (5.1)	467 (4.4)	134 (1.3)	356 (3.4)
NOVA 4 (*n* = 12,630)	2480 (19.6)	2105 (16.7)	220 (1.7)	25 (0.2)	62 (0.5)	73 (0.6)	685 (5.4)	561 (4.4)	256 (2.0)	66 (0.5)	258 (2.0)
Beverages * (*n* = 3377)	395 (11.7)	316 (9.4)	19 (0.6)	/	/	61 (1.8)	429 (12.7)	155 (4.6)	63 (1.9)	21 (0.6)	9 (0.3)
NOVA 1–3 (*n* = 1848)	55 (3.0)	46 (2.5)	9 (0.5)	/	/	/	386 (20.9)	114 (6.2)	50 (2.7)	21 (1.1)	8 (0.4)
NOVA 4 (*n* = 1529)	340 (22.2)	270 (17.7)	10 (0.7)	/	/	61 (4.0)	43 (2.8)	41 (2.7)	13 (0.9)	/	1 (0.1)
Bread and bakery products (*n* = 2393)	492 (20.6)	476 (19.9)	23 (1.0)	/	/	/	249 (10.4)	165 (6.9)	50 (2.1)	6 (0.3)	/
NOVA 1–3 (*n* = 407)	82 (20.1)	73 (17.9)	15 (3.7)	/	/	/	163 (40)	76 (18.7)	22 (5.4)	1 (0.2)	/
NOVA 4 (*n* = 1985)	410 (20.7)	403 (20.3)	8 (0.4)	/	/	/	86 (4.3)	89 (4.5)	28 (1.4)	5 (0.3)	/
Cereal and cereal products * (*n* = 2196)	398 (18.1)	266 (12.1)	114 (5.2)	9 (0.4)	1 (<0.1)	9 (0.4)	465 (21.2)	99 (4.5)	86 (3.9)	2 (0.1)	/
NOVA 1–3 (*n* = 1632)	209 (12.8)	154 (9.4)	42 (2.6)	9 (0.6)	1 (0.1)	3 (0.2)	425 (26)	61 (3.7)	79 (4.8)	2 (0.1)	/
NOVA 4 (*n* = 564)	189 (33.5)	112 (19.9)	72 (12.8)	/	/	6 (1.1)	40 (7.1)	38 (6.7)	7 (1.2)	/	/
Confectionery * (*n* = 2463)	438 (17.8)	427 (17.3)	10 (0.4)	/	/	1 (<0.1)	253 (10.3)	108 (4.4)	11 (0.4)	/	/
NOVA 1–3 (*n* = 183)	7 (3.8)	7 (3.8)	/	/	/	/	127 (69.4)	40 (21.9)	3 (1.6)	/	/
NOVA 4 (*n* = 2280)	431 (18.9)	420 (18.4)	10 (0.4)	/	/	1 (<0.1)	126 (5.5)	68 (3.0)	8 (0.4)	/	/
Convenience foods * (*n* = 1067)	268 (25.1)	234 (21.9)	31 (2.9)	/	/	3 (0.3)	89 (8.3)	52 (4.9)	12 (1.1)	/	2 (0.2)
NOVA 1–3 (*n* = 275)	41 (14.9)	34 (12.4)	5 (1.8)	/	/	2 (0.7)	46 (16.7)	18 (6.5)	6 (2.2)	/	1 (0.4)
NOVA 4 (*n* = 792)	227 (28.7)	200 (25.3)	26 (3.3)	/	/	1 (0.1)	43 (5.4)	34 (4.3)	6 (0.8)	/	1 (0.1)
Dairy and alternatives * (*n* = 3459)	690 (19.9)	566 (16.4)	17 (0.5)	38 (1.1)	67 (1.9)	3 (0.1)	427 (12.3)	216 (6.2)	304 (8.8)	34 (1.0)	483 (14.0)
NOVA 1–3 (*n* = 1408)	188 (13.4)	161 (11.4)	6 (0.4)	15 (1.1)	5 (0.4)	/	263 (18.7)	59 (4.2)	155 (11)	28 (2.0)	276 (19.6)
NOVA 4 (*n* = 2051)	502 (24.5)	405 (19.7)	11 (0.5)	23 (1.1)	62 (3.0)	3 (0.1)	164 (8.0)	157 (7.7)	149 (7.3)	6 (0.3)	207 (10.1)
Edible oils and emulsions (*n* = 609)	56 (9.2)	25 (4.1)	9 (1.5)	23 (3.8)	/	/	136 (22.3)	35 (5.7)	24 (3.9)	37 (6.1)	9 (1.5)
NOVA 1–3 (*n* = 548)	49 (8.9)	19 (3.5)	8 (1.5)	23 (4.2)	/	/	131 (23.9)	23 (4.2)	23 (4.2)	37 (6.8)	9 (1.6)
NOVA 4 (*n* = 61)	7 (11.5)	6 (9.8)	1 (1.6)	/	/	/	5 (8.2)	12 (19.7)	1 (1.6)	/	/
Eggs (*n* = 103) †	5 (4.9)	5 (4.9)	/	/	/	/	11 (10.7)	/	14 (13.6)	8 (7.8)	/
Fish and fish products (*n* = 563)	67 (11.9)	60 (10.7)	7 (1.2)	/	/	1 (0.2)	7 (1.2)	/	4 (0.7)	/	/
NOVA 1–3 (*n* = 405)	54 (13.3)	47 (11.6)	7 (1.7)	/	/	1 (0.2)	6 (1.5)	/	3 (0.7)	/	/
NOVA 4 (*n* = 158)	13 (8.2)	13 (8.2)	/	/	/	/	1 (0.6)	/	1 (0.6)	/	/
Fruit and vegetables * (*n* = 2629)	218 (8.3)	170 (6.5)	49 (1.9)	/	/	/	476 (18.1)	58 (2.2)	65 (2.5)	14 (0.5)	2 (0.1)
NOVA 1–3 (*n* = 2175)	193 (8.9)	151 (6.9)	43 (2.0)	/	/	/	432 (19.9)	55 (2.5)	60 (2.8)	14 (0.6)	2 (0.1)
NOVA 4 (*n* = 454)	25 (5.5)	19 (4.2)	6 (1.3)	/	/	/	44 (9.7)	3 (0.7)	5 (1.1)	/	/
Meat, meat products and alternatives * (*n* = 2124)	130 (6.1)	127 (6)	1 (<0.1)	2 (0.1)	/	/	89 (4.2)	49 (2.3)	40 (1.9)	74 (3.5)	109 (5.1)
NOVA 1–3 (*n* = 487)	13 (2.7)	13 (2.7)	/	/	/	/	36 (7.4)	18 (3.7)	21 (4.3)	19 (3.9)	60 (12.3)
NOVA 4 (*n* = 1637)	117 (7.1)	114 (7.0)	1 (0.1)	2 (0.1)	/	/	53 (3.2)	31 (1.9)	19 (1.2)	55 (3.4)	49 (3.0)
Snack foods * (*n* = 619)	193 (31.2)	99 (16.0)	93 (15.0)	/	/	1 (0.2)	60 (9.7)	54 (8.7)	5 (0.8)	/	/
NOVA 1–3 (*n* = 256)	56 (21.9)	34 (13.3)	22 (8.6)	/	/	/	53 (20.7)	24 (9.4)	5 (2.0)	/	/
NOVA 4 (*n* = 363)	137 (37.7)	65 (17.9)	71 (19.6)	/	/	1 (0.3)	7 (1.9)	30 (8.3)	/	/	/
Sauces and spreads * (*n* = 1296)	117 (9.0)	110 (8.5)	5 (0.4)	/	/	3 (0.2)	207 (16.0)	107 (8.3)	33 (2.5)	/	/
NOVA 1–3 (*n* = 553)	36 (6.5)	32 (5.8)	2 (0.4)	/	/	3 (0.5)	134 (24.2)	49 (8.9)	14 (2.5)	/	/
NOVA 4 (*n* = 743)	81 (10.9)	78 (10.5)	3 (0.4)	/	/	/	73 (9.8)	58 (7.8)	19 (2.6)	/	/
Sugar, honey and related products (*n* = 276)	7 (2.5)	6 (2.2)	1 (0.4)	/	/	/	52 (18.8)	1 (0.4)	12 (4.3)	4 (1.4)	/
NOVA 1–3 (*n* = 263)	6 (2.3)	6 (2.3)	/	/	/	/	52 (19.8)	1 (0.4)	12 (4.6)	4 (1.5)	/
NOVA 4 (*n* = 13)	1 (7.7)	/	1 (7.7)	/	/	/	/	/	/	/	/

RI-Energy, Reference Intakes displaying only energy value; RI Full, Nutrient-specific Reference Intakes; PH, Protect Health Symbol; NS, Nutri-Score; MTL, Multiple Traffic Light System; Non-GMO: non-genetically modified organisms; Other: other food quality schemes (displayed on Figure 1); SQ, Selected Quality—Slovenia. * Categories with statistically significant differences in the prevalence of FOPNL between NOVA 4 and NOVA 1–3 using z-test (*p* < 0.05). † All products were NOVA 1–3. ‡ Not included in the table, as they appeared only on individual products, are the Healthy Choice (three Convenience foods) and the Keyhole (two Cereal and cereal products and one Dairy product).

## Data Availability

The raw data supporting the conclusions of this article will be made available by the authors without undue reservation (without disclosure of specific brands or retailers).

## Supplementary Materials

The following supporting information can be downloaded at: https://www.mdpi.com/article/10.3390/nu17101731/s1, Table S1: Distribution of NOVA classification groups across food categories and subcategories in the 2020 Slovenian food supply (*n*= 23,173); Table S2: Prevalence of Front-of-Package Nutrition Labelling (FOPNL) and Subjective Nutrition-Related Elements (SNRE) Across Subcategories and Processing Levels in the 2020 Slovenian Food Supply (*n* = 23,173).
