# Supplementary material for: Ultra-Processed Foods in the Food Supply: Prevalence, Nutritional Composition and Use of Voluntary Labelling Schemes
Source: Nutrients. 2025 May 20;17(10):1731. doi: 10.3390/nu17101731 (PMC12113896; doi:10.3390/nu17101731)
Supplement: Supplementary file 1 [file nutrients-17-01731-s001.zip › nutrients-3619457-supplementary.pdf]

Supplementary Material Table S1: Distribution of NOVA classification groups across food categories and subcategories in the 2020 Slovenian food supply (*n*= 23,173).

	NOVA (n (%))			
	**1**	**2**	**3**	**4**
**TOTAL (*n*= 23,173)**	**5130 (22.1)**	**913 (3.9)**	**4500 (19.4)**	**12630 (54.5)**
**Beverages (*n*= 3377)**	**1645 (48.7)**	**/**	**203 (6)**	**1529 (45.3)**
Coffee (*n*= 650)	428 (65.8)	/	16 (2.5)	206 (31.7)
Cordials (*n*= 190)	5 (2.6)	/	21 (11.1)	164 (86.3)
Juices and nectars (*n*= 609)	373 (61.2)	/	138 (22.7)	98 (16.1)
Soft drinks (*n*= 1081)	/	/	24 (2.2)	1057 (97.8)
Tea (*n*= 659)	651 (98.8)	/	4 (0.6)	4 (0.6)
Waters (*n*= 188)	188 (100)	/	/	/
**Bread and bakery products (*n*= 2392)**	**7 (0.3)**	**/**	**400 (16.7)**	**1985 (83.0)**
Biscuits (*n*= 1122)	/	/	86 (7.7)	1036 (92.3)
Bread (*n*= 297)	4 (1.3)	/	61 (20.5)	232 (78.1)
Cakes, muffins and pastry (*n*= 639)	3 (0.5)	/	80 (12.5)	556 (87.0)
Crispy bread (*n*= 235)	/	/	157 (66.8)	78 (33.2)
Other bakery products (*n*= 99)	/	/	16 (16.2)	83 (83.8)
**Cereal and cereal products (*n*= 2196)**	**1387 (63.2)**	**/**	**245 (11.2)**	**564 (25.7)**
Breakfast cereals (*n*= 487)	19 (3.9)	/	129 (26.5)	339 (69.6)
Cereal bars (*n*= 71)	/	/	3 (4.2)	68 (95.8)
Cereal flakes and bran (*n*= 63)	63 (100)	/	/	/
Dry pasta (*n*= 797)	682 (85.6)	/	66 (8.3)	49 (6.1)
Fresh filled pasta (*n*= 143)	/	/	40 (28.0)	103 (72.0)
Fresh pasta (*n*= 24)	20 (83.3)	/	2 (8.3)	2 (8.3)
Unprocessed cereals (*n*= 611)	603 (98.7)	/	5 (0.8)	3 (0.5)
**Confectionery (*n*= 2463)**	**7 (0.3)**	**/**	**176 (7.1)**	**2280 (92.6)**
Chewing gum (*n*= 139)	/	/	/	139 (100)
Chocolate and sweets (*n*= 2166)	7 (0.3)	/	176 (8.1)	1983 (91.6)
Jelly candy (*n*= 158)	/	/	/	158 (100)
**Convenience foods (*n*= 1067)**	**3 (0.3)**	**/**	**272 (25.5)**	**792 (74.2)**
Pizza (*n*= 72)	/	/	/	72 (100)
Pre-prepared salads and sandwiches (*n*= 129)	1 (0.8)	/	32 (24.8)	96 (74.4)
Ready meals (*n*= 385)	1 (0.3)	/	102 (26.5)	282 (73.2)
Side dishes (*n*= 224)	1 (0.4)	/	95 (42.4)	128 (57.1)
Soup (*n*= 257)	/	/	43 (16.7)	214 (83.3)
**Dairy (*n*= 3459)**	**499 (14.4)**	**/**	**909 (26.3)**	**2051 (59.3)**
Cheese and processed cheese (*n*= 843)	/	/	609 (72.2)	234 (27.8)
Cheese imitates (*n*= 34)	/	/	/	34 (100)
Cottage cheese (*n*= 83)	/	/	79 (95.2)	4 (4.8)
Cream (*n*= 154)	59 (38.3)	/	23 (14.9)	72 (46.8)
Cream imitates (*n*= 38)	1 (2.6)	/	/	37 (97.4)
Desserts (*n*= 298)	/	/	23 (7.7)	275 (92.3)
Flavoured yogurt (*n*= 585)	/	/	40 (6.8)	545 (93.2)
Ice cream and edible ices (*n*= 588)	7 (1.2)	/	6 (1.0)	575 (97.8)

	NOVA (n (%))			
	**1**	**2**	**3**	**4**
Milk and milk drinks (*n*= 324)	167 (51.5)	/	22 (6.8)	135 (41.7)
Milk alternatives (*n*= 185)	/	/	99 (53.5)	86 (46.5)
Plain yogurt (*n*= 285)	263 (92.3)	/	2 (0.7)	20 (7.0)
Yogurt alternatives (*n*= 42)	2 (4.8)	/	6 (14.3)	34 (81.0)
**Edible oils and emulsions (*n*= 609)**	**/**	**547 (89.8)**	**1 (0.2)**	**61 (10)**
Butter (*n*= 72)	/	68 (94.4)	1 (1.4)	3 (4.2)
Cooking oils (*n*= 485)	/	477 (98.4)	/	8 (1.6)
Margarine (*n*= 52)	/	2 (3.8)	/	50 (96.2)
**Eggs (*n*= 103)**	**103 (100)**	**/**	**/**	**/**
**Fish and fish products (*n*= 563)**	**90 (16.0)**	**/**	**315 (56.0)**	**158 (28.1)**
Canned fish (*n*= 295)	/	/	229 (77.6)	66 (22.4)
Processed fish products (*n*= 144)	2 (1.4)	/	67 (46.5)	75 (52.1)
Unprocessed fish (*n*= 124)	88 (71.0)	/	19 (15.3)	17 (13.7)
**Fruit, vegetables and nuts (*n*= 2629)**	**1147 (43.6)**	**/**	**1028 (39.1)**	**454 (17.3)**
Canned fruit (*n*= 111)	/	/	77 (69.4)	34 (30.6)
Canned vegetables (*n*= 734)	3 (0.4)	/	663 (90.3)	68 (9.3)
Dried fruit (*n*= 287)	169 (58.9)	/	92 (32.1)	26 (9.1)
Dried vegetables (*n*= 104)	100 (96.2)	/	4 (3.8)	/
Fresh fruit (*n*= 113)	113 (100)	/	/	/
Fresh vegetables (*n*= 248)	243 (98.0)	/	5 (2.0)	/
Frozen fruit (*n*= 48)	48 (100)	/	/	/
Frozen vegetables (*n*= 136)	121 (89.0)	/	10 (7.4)	5 (3.7)
Jam and spreads (*n*= 321)	/	/	26 (8.1)	295 (91.9)
Nuts and fruit mixes (*n*= 94)	31 (33.0)	/	48 (51.1)	15 (16.0)
Nuts and seeds (*n*= 433)	319 (73.7)	/	103 (23.8)	11 (2.5)
**Meat, meat products and alternatives (*n*= 2124)**	**231 (10.9)**	**65 (3.1)**	**191 (9.0)**	**1637 (77.1)**
Animal fat products (*n*= 81)	/	65 (80.2)	12 (14.8)	4 (4.9)
Meat alternatives (*n*= 165)	6 (3.6)	/	53 (32.1)	106 (64.2)
Processed meat (*n*= 1586)	2 (0.1)	/	94 (5.9)	1490 (93.9)
Unprocessed meat (*n*= 292)	223 (76.4)	/	32 (11.0)	37 (12.7)
**Snack foods (*n*= 619)**	**8 (1.3)**	**/**	**248 (40.1)**	**363 (58.6)**
**Sauces and spreads (*n*= 1296)**	**3 (0.2)**	**37 (2.9)**	**513 (39.6)**	**743 (57.3)**
Mayonnaise and dressings (*n*= 109)	/	/	24 (22.0)	85 (78.0)
Nut spreads (*n*= 68)	/	32 (47.1)	19 (27.9)	17 (25.0)
Spreads (*n*= 171)	/	4 (2.3)	86 (50.3)	81 (47.4)
Sauces (*n*= 847)	3 (0.4)	1 (0.1)	378 (44.6)	465 (54.9)
Sweet spreads (*n*= 101)	/	/	6 (5.9)	95 (94.1)
**Sugars, honey and related products (*n*= 276)**	**/**	**263 (95.3)**	**/**	**13 (4.7)**
Honey (*n*= 127)	/	126 (99.2)	/	1 (0.8)
Sugar (*n*= 108)	/	108 (100)	/	/
Syrup (*n*= 41)	/	29 (70.7)	/	12 (29.3)

Supplementary Material Table S2: Prevalence of Front-of-Package Nutrition Labelling (FOPNL) and Subjective Nutrition-Related Elements (SNRE) Across Subcategories and Processing Levels in the 2020 Slovenian Food Supply (*n* = 23,173).

		FOPNL (n (%))‡						SNRE (n (%))				
		**Any FOPNL**	**RI-Energy**	**RI-Full**	**PH**	**NS**	**MTL**	**Organic**	**Vegan**	**Non-GMO**	**Other**	**SQ**
**BEVERAGES**	Juices and nectars* (*n*= 609)	78 (12.8)	69 (11.3)	9 (1.5)	/	/	/	146 (24)	120 (19.7)	22 (3.6)	5 (0.8)	8 (1.3)
	NOVA 1-3 (*n*= 511)	54 (10.6)	45 (8.8)	9 (1.8)	/	/	/	142 (27.8)	110 (21.5)	22 (4.3)	5 (1)	8 (1.6)
	NOVA 4 (*n*= 98)	24 (24.5)	24 (24.5)	/	/	/	/	4 (4.1)	10 (10.2)	/	/	/
	Soft drinks (*n*= 1081)	240 (22.2)	181 (16.7)	1 (0.1)	/	/	59 (5.5)	32 (3)	23 (2.1)	4 (0.4)	/	/
	NOVA 1-3 (*n*= 24)	/	/	/	/	/	/	5 (20.8)	1 (4.2)	/	/	/
	NOVA 4 (*n*= 1057)	240 (22.7)	181 (17.1)	1 (0.1)	/	/	59 (5.6)	27 (2.6)	22 (2.1)	4 (0.4)	/	/
	Cordials (*n*= 190)	12 (6.3)	12 (6.3)	/	/	/	/	17 (8.9)	5 (2.6)	4 (2.1)	/	/
	NOVA 1-3 (*n*= 26)	/	/	/	/	/	/	12 (46.2)	2 (7.7)	1 (3.8)	/	/
	NOVA 4 (*n*= 164)	12 (7.3)	12 (7.3)	/	/	/	/	5 (3)	3 (1.8)	3 (1.8)	/	/
	Tea (*n*= 659)	/	/	/	/	/	/	175 (26.6)	1 (0.2)	21 (3.2)	3 (0.5)	/
	NOVA 1-3 (*n*= 655)	/	/	/	/	/	/	175 (26.7)	1 (0.2)	21 (3.2)	3 (0.5)	/
	NOVA 4 (*n*= 4)	/	/	/	/	/	/	/	/	/	/	/
	Coffee (*n*= 650)	65 (10)	54 (8.3)	9 (1.4)	/	/	2 (0.3)	59 (9.1)	6 (0.9)	12 (1.8)	/	1 (0.2)
	NOVA 1-3 (*n*= 444)	1 (0.2)	1 (0.2)	/	/	/	/	52 (11.7)	/	6 (1.4)	/	/
	NOVA 4 (*n*= 206)	64 (31.1)	53 (25.7)	9 (4.4)	/	/	2 (1)	7 (3.4)	6 (2.9)	6 (2.9)	/	1 (0.5)
	Waters (*n*= 188)	/	/	/	/	/	/	/	/	/	13 (6.9)	/
	NOVA 1-3 (*n*= 188)	/	/	/	/	/	/	/	/	/	13 (6.9)	/
**BREAD AND BAKERY PRODUCTS**	Bread (*n*= 297)	45 (15.2)	45 (15.2)	/	/	/	/	20 (6.7)	17 (5.7)	10 (3.4)	/	/
	NOVA 1-3 (*n*= 65)	4 (6.2)	4 (6.2)	/	/	/	/	9 (13.8)	12 (18.5)	3 (4.6)	/	/
	NOVA 4 (*n*= 232)	41 (17.7)	41 (17.7)	/	/	/	/	11 (4.7)	5 (2.2)	7 (3)	/	/
	Crispy bread* (*n*= 235)	71 (30.2)	60 (25.5)	16 (6.8)	/	/	/	46 (19.6)	32 (13.6)	11 (4.7)	/	/
	NOVA 1-3 (*n*= 157)	57 (36.3)	48 (30.6)	14 (8.9)	/	/	/	46 (29.3)	24 (15.3)	11 (7)	/	/
	NOVA 4 (*n*= 78)	14 (17.9)	12 (15.4)	2 (2.6)	/	/	/	/	8 (10.3)	/	/	/
	Other (*n*= 99)	11 (11.1)	9 (9.1)	2 (2)	/	/	/	2 (2)	21 (21.2)	1 (1)	/	/
	NOVA 1-3 (*n*= 16)	2 (12.5)	2 (12.5)	/	/	/	/	/	4 (25)	/	/	/
	NOVA 4 (*n*= 83)	9 (10.8)	7 (8.4)	2 (2.4)	/	/	/	2 (2.4)	17 (20.5)	1 (1.2)	/	/
	Biscuits (*n*= 1122)	230 (20.5)	227 (20.2)	4 (0.4)	/	/	/	111 (9.9)	67 (6)	15 (1.3)	1 (0.1)	/
	NOVA 1-3 (*n*= 86)	4 (4.7)	4 (4.7)	/	/	/	/	58 (67.4)	16 (18.6)	1 (1.2)	/	/
	NOVA 4 (*n*= 1036)	226 (21.8)	223 (21.5)	4 (0.4)	/	/	/	53 (5.1)	51 (4.9)	14 (1.4)	1 (0.1)	/
	Cakes, muffins & pastry (*n*= 639)	135 (21.1)	135 (21.1)	1 (0.2)	/	/	/	70 (11)	28 (4.4)	13 (2)	5 (0.8)	/
	NOVA 1-3 (*n*= 83)	15 (18.1)	15 (18.1)	1 (1.2)	/	/	/	50 (60.2)	20 (24.1)	7 (8.4)	1 (1.2)	/
	NOVA 4 (*n*= 556)	120 (21.6)	120 (21.6)	/	/	/	/	20 (3.6)	8 (1.4)	6 (1.1)	4 (0.7)	/

		FOPNL (n (%))‡						SNRE (n (%))				
		**Any FOPNL**	**RI-Energy**	**RI-Full**	**PH**	**NS**	**MTL**	**Organic**	**Vegan**	**Non-GMO**	**Other**	**SQ**
**CEREAL AND CEREAL PRODUCTS**	Cereal bars (*n*= 71)	15 (21.1)	15 (21.1)	/	/	/	/	2 (2.8)	3 (4.2)	2 (2.8)	/	/
	NOVA 1-3 (*n*= 3)	/	/	/	/	/	/	1 (33.3)	/	/	/	/
	NOVA 4 (*n*= 68)	15 (22.1)	15 (22.1)	/	/	/	/	1 (1.5)	3 (4.4)	2 (2.9)	/	/
	Breakfast cereals* (*n*= 487)	204 (41.9)	104 (21.4)	92 (18.9)	1 (0.2)	1 (0.2)	8 (1.6)	108 (22.2)	45 (9.2)	12 (2.5)	/	/
	NOVA 1-3 (*n*= 148)	45 (30.4)	22 (14.9)	20 (13.5)	1 (0.7)	1 (0.7)	2 (1.4)	73 (49.3)	18 (12.2)	7 (4.7)	/	/
	NOVA 4 (*n*= 339)	159 (46.9)	82 (24.2)	72 (21.2)	/	/	6 (1.8)	35 (10.3)	27 (8)	5 (1.5)	/	/
	Cereal flakes and bran (*n*= 63)	8 (12.7)	7 (11.1)	1 (1.6)	/	/	/	42 (66.7)	1 (1.6)	10 (15.9)	/	/
	NOVA 1-3 (*n*= 63)	8 (12.7)	7 (11.1)	1 (1.6)	/	/	/	42 (66.7)	1 (1.6)	10 (15.9)	/	/
	Dry pasta (*n*= 797)	113 (14.2)	102 (12.8)	2 (0.3)	8 (1)	/	/	119 (14.9)	28 (3.5)	21 (2.6)	/	/
	NOVA 1-3 (*n*= 748)	107 (14.3)	96 (12.8)	2 (0.3)	8 (1.1)	/	/	119 (15.9)	25 (3.3)	21 (2.8)	/	/
	NOVA 4 (*n*= 49)	6 (12.2)	6 (12.2)	/	/	/	/	/	3 (6.1)	/	/	/
	Fresh pasta (*n*= 24)	7 (29.2)	3 (12.5)	4 (16.7)	/	/	/	4 (16.7)	2 (8.3)	/	/	/
	NOVA 1-3 (*n*= 22)	6 (27.3)	2 (9.1)	4 (18.2)	/	/	/	4 (18.2)	2 (9.1)	/	/	/
	NOVA 4 (*n*= 2)	1 (50)	1 (50)	/	/	/	/	/	/	/	/	/
	Fresh filled pasta (*n*= 143)	11 (7.7)	9 (6.3)	2 (1.4)	/	/	/	7 (4.9)	5 (3.5)	/	2 (1.4)	/
	NOVA 1-3 (*n*= 40)	3 (7.5)	1 (2.5)	2 (5)	/	/	/	3 (7.5)	/	/	2 (5)	/
	NOVA 4 (*n*= 103)	8 (7.8)	8 (7.8)	/	/	/	/	4 (3.9)	5 (4.9)	/	/	/
	Unprocessed cereals (*n*= 611)	40 (6.5)	26 (4.3)	13 (2.1)	/	/	1 (0.2)	183 (30)	15 (2.5)	41 (6.7)	/	/
	NOVA 1-3 (*n*= 608)	40 (6.6)	26 (4.3)	13 (2.1)	/	/	1 (0.2)	183 (30.1)	15 (2.5)	41 (6.7)	/	/
	NOVA 4 (*n*= 3)	/	/	/	/	/	/	/	/	/	/	/
**CONFECTIONERY**	Chocolate and sweets* (*n*= 2166)	420 (19.4)	409 (18.9)	10 (0.5)	/	/	1 (0)	239 (11)	93 (4.3)	11 (0.5)	/	/
	NOVA 1-3 (*n*= 183)	7 (3.8)	7 (3.8)	/	/	/	/	127 (69.4)	40 (21.9)	3 (1.6)	/	/
	NOVA 4 (*n*= 1983)	413 (20.8)	402 (20.3)	10 (0.5)	/	/	1 (0.1)	112 (5.6)	53 (2.7)	8 (0.4)	/	/
	Jelly candy (*n*= 158)	13 (8.2)	13 (8.2)	/	/	/	/	14 (8.9)	15 (9.5)	/	/	/
	NOVA 4 (*n*= 158)	13 (8.2)	13 (8.2)	/	/	/	/	14 (8.9)	15 (9.5)	/	/	/
	Chewing gum (*n*= 139)	5 (3.6)	5 (3.6)	/	/	/	/	/	/	/	/	/
	NOVA 4 (*n*= 139)	5 (3.6)	5 (3.6)	/	/	/	/	/	/	/	/	/
**CONVENIENCE FOODS**	Pizza (*n*= 72)	32 (44.4)	32 (44.4)	/	/	/	/	/	8 (11.1)	/	/	1 (1.4)
	NOVA 4 (*n*= 72)	32 (44.4)	32 (44.4)	/	/	/	/	/	8 (11.1)	/	/	1 (1.4)
	Soup* (*n*= 257)	101 (39.3)	83 (32.3)	18 (7)	/	/	/	59 (23)	15 (5.8)	1 (0.4)	/	/
	NOVA 1-3 (*n*= 43)	7 (16.3)	7 (16.3)	/	/	/	/	27 (62.8)	6 (14)	/	/	/
	NOVA 4 (*n*= 214)	94 (43.9)	76 (35.5)	18 (8.4)	/	/	/	32 (15)	9 (4.2)	1 (0.5)	/	/
	Ready meals (*n*= 385)	88 (22.9)	77 (20)	8 (2.1)	/	/	3 (0.8)	23 (6)	17 (4.4)	8 (2.1)	/	/
	NOVA 1-3 (*n*= 103)	18 (17.5)	11 (10.7)	5 (4.9)	/	/	2 (1.9)	14 (13.6)	9 (8.7)	5 (4.9)	/	/
	NOVA 4 (*n*= 282)	70 (24.8)	66 (23.4)	3 (1.1)	/	/	1 (0.4)	9 (3.2)	8 (2.8)	3 (1.1)	/	/

		FOPNL (n (%))‡						SNRE (n (%))				
		Any FOPNL	RI-Energy	RI-Full	PH	NS	MTL	Organic	Vegan	Non-GMO	Other	SQ
	Pre-prepared salads and sandwiches (*n*= 129)	15 (11.6)	15 (11.6)	/	/	/	/	/	/	/	/	/
	NOVA 1-3 (*n*= 33)	2 (6.1)	2 (6.1)	/	/	/	/	/	/	/	/	/
	NOVA 4 (*n*= 96)	13 (13.5)	13 (13.5)	/	/	/	/	/	/	/	/	/
	Side dishes (*n*= 224)	32 (14.3)	27 (12.1)	5 (2.2)	/	/	/	7 (3.1)	12 (5.4)	3 (1.3)	/	1 (0.4)
	NOVA 1-3 (*n*= 96)	14 (14.6)	14 (14.6)	/	/	/	/	5 (5.2)	3 (3.1)	1 (1)	/	1 (1)
	NOVA 4 (*n*= 128)	18 (14.1)	13 (10.2)	5 (3.9)	/	/	/	2 (1.6)	9 (7)	2 (1.6)	/	/
**DAIRY AND ALTERNATIVES**	Cottage cheese (*n*= 83)	13 (15.7)	10 (12)	1 (1.2)	2 (2.4)	/	/	7 (8.4)	/	12 (14.5)	1 (1.2)	27 (32.5)
	NOVA 1-3 (*n*= 79)	13 (16.5)	10 (12.7)	1 (1.3)	2 (2.5)	/	/	7 (8.9)	/	12 (15.2)	1 (1.3)	27 (34.2)
	NOVA 4 (*n*= 4)	/	/	/	/	/	/	/	/	/	/	/
	Cheese and processed cheese (*n*= 843)	111 (13.2)	107 (12.7)	4 (0.5)	/	/	/	67 (7.9)	10 (1.2)	70 (8.3)	31 (3.7)	128 (15.2)
	NOVA 1-3 (*n*= 609)	72 (11.8)	69 (11.3)	3 (0.5)	/	/	/	66 (10.8)	10 (1.6)	58 (9.5)	25 (4.1)	99 (16.3)
	NOVA 4 (*n*= 234)	39 (16.7)	38 (16.2)	1 (0.4)	/	/	/	1 (0.4)	/	12 (5.1)	6 (2.6)	29 (12.4)
	Cheese alternatives (*n*= 34)	/	/	/	/	/	/	11 (32.4)	27 (79.4)	/	/	/
	NOVA 4 (*n*= 34)	/	/	/	/	/	/	11 (32.4)	27 (79.4)	/	/	/
	Plain yogurt (*n*= 285)	43 (15.1)	29 (10.2)	1 (0.4)	12 (4.2)	1 (0.4)	/	38 (13.3)	8 (2.8)	44 (15.4)	2 (0.7)	89 (31.2)
	NOVA 1-3 (*n*= 265)	35 (13.2)	21 (7.9)	1 (0.4)	12 (4.5)	1 (0.4)	/	38 (14.3)	8 (3)	41 (15.5)	2 (0.8)	86 (32.5)
	NOVA 4 (*n*= 20)	8 (40)	8 (40)	/	/	/	/	/	/	3 (15)	/	3 (15)
	Flavoured yogurt (*n*= 585)	151 (25.8)	84 (14.4)	/	23 (3.9)	44 (7.5)	/	66 (11.3)	6 (1)	99 (16.9)	/	134 (22.9)
	NOVA 1-3 (*n*= 40)	8 (20)	5 (12.5)	/	/	3 (7.5)	/	9 (22.5)	1 (2.5)	3 (7.5)	/	8 (20)
	NOVA 4 (*n*= 545)	143 (26.2)	79 (14.5)	/	23 (4.2)	41 (7.5)	/	57 (10.5)	5 (0.9)	96 (17.6)	/	126 (23.1)
	Yogurt alternatives (*n*= 42)	4 (9.5)	/	/	/	4 (9.5)	/	20 (47.6)	13 (31)	/	/	/
	NOVA 1-3 (*n*= 8)	/	/	/	/	/	/	6 (75)	2 (25)	/	/	/
	NOVA 4 (*n*= 34)	4 (11.8)	/	/	/	4 (11.8)	/	14 (41.2)	11 (32.4)	/	/	/
	Milk and milk drinks (*n*= 324)	53 (16.4)	41 (12.7)	6 (1.9)	1 (0.3)	4 (1.2)	1 (0.3)	32 (9.9)	1 (0.3)	21 (6.5)	/	59 (18.2)
	NOVA 1-3 (*n*= 189)	29 (15.3)	27 (14.3)	1 (0.5)	1 (0.5)	/	/	29 (15.3)	1 (0.5)	20 (10.6)	/	50 (26.5)
	NOVA 4 (*n*= 135)	24 (17.8)	14 (10.4)	5 (3.7)	/	4 (3)	1 (0.7)	3 (2.2)	/	1 (0.7)	/	9 (6.7)
	Milk alternatives* (*n*= 185)	44 (23.8)	42 (22.7)	/	/	1 (0.5)	/	118 (63.8)	84 (45.4)	6 (3.2)	/	/
	NOVA 1-3 (*n*= 99)	17 (17.2)	16 (16.2)	/	/	/	/	87 (87.9)	35 (35.4)	6 (6.1)	/	/
	NOVA 4 (*n*= 86)	27 (31.4)	26 (30.2)	/	/	1 (1.2)	/	31 (36)	49 (57)	/	/	/
	Cream (*n*= 154)	18 (11.7)	18 (11.7)	/	/	/	/	6 (3.9)	/	26 (16.9)	/	8 (5.2)
	NOVA 1-3 (*n*= 82)	10 (12.2)	10 (12.2)	/	/	/	/	5 (6.1)	/	11 (13.4)	/	5 (6.1)
	NOVA 4 (*n*= 72)	8 (11.1)	8 (11.1)	/	/	/	/	1 (1.4)	/	15 (20.8)	/	3 (4.2)
	Cream alternatives (*n*= 38)	8 (21.1)	8 (21.1)	/	/	/	1 (2.6)	20 (52.6)	9 (23.7)	3 (7.9)	/	/
	NOVA 1-3 (*n*= 1)	/	/	/	/	/	/	/	1 (100)	/	/	/
	NOVA 4 (*n*= 37)	8 (21.6)	8 (21.6)	/	/	/	1 (2.7)	20 (54.1)	8 (21.6)	3 (8.1)	/	/

		FOPNL (n (%))‡						SNRE (n (%))				
		**Any FOPNL**	**RI-Energy**	**RI-Full**	**PH**	**NS**	**MTL**	**Organic**	**Vegan**	**Non-GMO**	**Other**	**SQ**
	Desserts (*n*= 298)	51 (17.1)	37 (12.4)	2 (0.7)	/	13 (4.4)	/	21 (7)	10 (3.4)	2 (0.7)	/	5 (1.7)
	NOVA 1-3 (*n*= 23)	4 (17.4)	3 (13)	/	/	1 (4.3)	/	13 (56.5)	1 (4.3)	1 (4.3)	/	1 (4.3)
	NOVA 4 (*n*= 275)	47 (17.1)	34 (12.4)	2 (0.7)	/	12 (4.4)	/	8 (2.9)	9 (3.3)	1 (0.4)	/	4 (1.5)
	Ice cream and edible ices (*n*= 588)	194 (33)	190 (32.3)	3 (0.5)	/	/	1 (0.2)	21 (3.6)	48 (8.2)	21 (3.6)	/	33 (5.6)
	NOVA 1-3 (*n*= 13)	/	/	/	/	/	/	3 (23.1)	/	3 (23.1)	/	/
	NOVA 4 (*n*= 575)	194 (33.7)	190 (33)	3 (0.5)	/	/	1 (0.2)	18 (3.1)	48 (8.3)	18 (3.1)	/	33 (5.7)
**EDIBLE OILS AND EMULSIONS**	Butter (*n*= 72)	4 (5.6)	4 (5.6)	/	/	/	/	8 (11.1)	/	9 (12.5)	/	9 (12.5)
	NOVA 1-3 (*n*= 69)	4 (5.8)	4 (5.8)	/	/	/	/	8 (11.6)	/	8 (11.6)	/	9 (13)
	NOVA 4 (*n*= 3)	/	/	/	/	/	/	/	/	1 (33.3)	/	/
	Margarine (*n*= 52)	6 (11.5)	5 (9.6)	1 (1.9)	/	/	/	5 (9.6)	9 (17.3)	/	/	/
	NOVA 1-3 (*n*= 2)	/	/	/	/	/	/	/	/	/	/	/
	NOVA 4 (*n*= 50)	6 (12)	5 (10)	1 (2)	/	/	/	5 (10)	9 (18)	/	/	/
	Cooking oils (*n*= 485)	46 (9.5)	16 (3.3)	8 (1.6)	23 (4.7)	/	/	123 (25.4)	26 (5.4)	15 (3.1)	37 (7.6)	/
	NOVA 1-3 (*n*= 477)	45 (9.4)	15 (3.1)	8 (1.7)	23 (4.8)	/	/	123 (25.8)	23 (4.8)	15 (3.1)	37 (7.8)	/
	NOVA 4 (*n*= 8)	1 (12.5)	1 (12.5)	/	/	/	/	/	3 (37.5)	/	/	/
**EGGS**	Eggs (*n*= 103)	5 (4.9)	5 (4.9)	/	/	/	/	11 (10.7)	/	14 (13.6)	8 (7.8)	/
	NOVA 1-3 (*n*= 103)	5 (4.9)	5 (4.9)	/	/	/	/	11 (10.7)	/	14 (13.6)	8 (7.8)	/
**FISH AND FISH PRODUCTS**	Canned fish (*n*= 295)	16 (5.4)	15 (5.1)	1 (0.3)	/	/	/	/	/	/	/	/
	NOVA 1-3 (*n*= 229)	16 (7)	15 (6.6)	1 (0.4)	/	/	/	/	/	/	/	/
	NOVA 4 (*n*= 66)	/	/	/	/	/	/	/	/	/	/	/
	Processed fish products (*n*= 144)	8 (5.6)	7 (4.9)	/	/	/	1 (0.7)	4 (2.8)	/	1 (0.7)	/	/
	NOVA 1-3 (*n*= 69)	5 (7.2)	4 (5.8)	/	/	/	1 (1.4)	4 (5.8)	/	1 (1.4)	/	/
	NOVA 4 (*n*= 75)	3 (4)	3 (4)	/	/	/	/	/	/	/	/	/
	Unprocessed fish (*n*= 124)	43 (34.7)	38 (30.6)	6 (4.8)	/	/	/	3 (2.4)	/	3 (2.4)	/	/
	NOVA 1-3 (*n*= 107)	33 (30.8)	28 (26.2)	6 (5.6)	/	/	/	2 (1.9)	/	2 (1.9)	/	/
	NOVA 4 (*n*= 17)	10 (58.8)	10 (58.8)	/	/	/	/	1 (5.9)	/	1 (5.9)	/	/
**FRUIT, VEGETABLES AND NUTS**	Frozen vegetables (*n*= 136)	40 (29.4)	24 (17.6)	16 (11.8)	/	/	/	12 (8.8)	/	2 (1.5)	/	/
	NOVA 1-3 (*n*= 131)	38 (29)	22 (16.8)	16 (12.2)	/	/	/	12 (9.2)	/	2 (1.5)	/	/
	NOVA 4 (*n*= 5)	2 (40)	2 (40)	/	/	/	/	/	/	/	/	/
	Canned vegetables (*n*= 734)	17 (2.3)	15 (2)	2 (0.3)	/	/	/	98 (13.4)	10 (1.4)	13 (1.8)	10 (1.4)	/
	NOVA 1-3 (*n*= 666)	16 (2.4)	14 (2.1)	2 (0.3)	/	/	/	97 (14.6)	10 (1.5)	12 (1.8)	10 (1.5)	/
	NOVA 4 (*n*= 68)	1 (1.5)	1 (1.5)	/	/	/	/	1 (1.5)	/	1 (1.5)	/	/
	Dried vegetables (*n*= 104)	15 (14.4)	15 (14.4)	/	/	/	/	24 (23.1)	/	/	/	/
	NOVA 1-3 (*n*= 104)	15 (14.4)	15 (14.4)	/	/	/	/	24 (23.1)	/	/	/	/

		FOPNL (n (%))‡						SNRE (n (%))				
		**Any FOPNL**	**RI-Energy**	**RI-Full**	**PH**	**NS**	**MTL**	**Organic**	**Vegan**	**Non-GMO**	**Other**	**SQ**
	Fresh vegetables (*n*= 248)	1 (0.4)	1 (0.4)	/	/	/	/	46 (18.5)	/	3 (1.2)	4 (1.6)	/
	NOVA 1-3 (*n*= 248)	1 (0.4)	1 (0.4)	/	/	/	/	46 (18.5)	/	3 (1.2)	4 (1.6)	/
	Frozen fruit (*n*= 48)	7 (14.6)	4 (8.3)	3 (6.3)	/	/	/	4 (8.3)	/	1 (2.1)	/	/
	NOVA 1-3 (*n*= 48)	7 (14.6)	4 (8.3)	3 (6.3)	/	/	/	4 (8.3)	/	1 (2.1)	/	/
	Canned fruit (*n*= 111)	8 (7.2)	5 (4.5)	3 (2.7)	/	/	/	10 (9)	3 (2.7)	8 (7.2)	/	/
	NOVA 1-3 (*n*= 77)	5 (6.5)	3 (3.9)	2 (2.6)	/	/	/	8 (10.4)	3 (3.9)	7 (9.1)	/	/
	NOVA 4 (*n*= 34)	3 (8.8)	2 (5.9)	1 (2.9)	/	/	/	2 (5.9)	/	1 (2.9)	/	/
	Fresh fruit (*n*= 113)	/	/	/	/	/	/	40 (35.4)	/	2 (1.8)	/	1 (0.9)
	NOVA 1-3 (*n*= 113)	/	/	/	/	/	/	40 (35.4)	/	2 (1.8)	/	1 (0.9)
	Dried fruit (*n*= 287)	33 (11.5)	28 (9.8)	5 (1.7)	/	/	/	74 (25.8)	20 (7)	11 (3.8)	/	/
	NOVA 1-3 (*n*= 261)	33 (12.6)	28 (10.7)	5 (1.9)	/	/	/	71 (27.2)	19 (7.3)	11 (4.2)	/	/
	NOVA 4 (*n*= 26)	/	/	/	/	/	/	3 (11.5)	1 (3.8)	/	/	/
	Nuts and fruit mixes (*n*= 94)	12 (12.8)	9 (9.6)	3 (3.2)	/	/	/	20 (21.3)	16 (17)	3 (3.2)	/	/
	NOVA 1-3 (*n*= 79)	11 (13.9)	8 (10.1)	3 (3.8)	/	/	/	20 (25.3)	16 (20.3)	3 (3.8)	/	/
	NOVA 4 (*n*= 15)	1 (6.7)	1 (6.7)	/	/	/	/	/	/	/	/	/
	Jam and spreads (*n*= 321)	15 (4.7)	11 (3.4)	4 (1.2)	/	/	/	38 (11.8)	1 (0.3)	3 (0.9)	/	/
	NOVA 1-3 (*n*= 26)	/	/	/	/	/	/	/	/	/	/	/
	NOVA 4 (*n*= 295)	15 (5.1)	11 (3.7)	4 (1.4)	/	/	/	38 (12.9)	1 (0.3)	3 (1)	/	/
	Nuts and seeds (*n*= 433)	70 (16.2)	58 (13.4)	13 (3)	/	/	/	110 (25.4)	8 (1.8)	19 (4.4)	/	1 (0.2)
	NOVA 1-3 (*n*= 422)	67 (15.9)	56 (13.3)	12 (2.8)	/	/	/	110 (26.1)	7 (1.7)	19 (4.5)	/	1 (0.2)
	NOVA 4 (*n*= 11)	3 (27.3)	2 (18.2)	1 (9.1)	/	/	/	/	1 (9.1)	/	/	/
**MEAT, MEAT PRODUCTS AND ALTERNATIVES**	Unprocessed meat (*n*= 292)	4 (1.4)	4 (1.4)	/	/	/	/	7 (2.4)	/	17 (5.8)	7 (2.4)	71 (24.3)
	NOVA 1-3 (*n*= 255)	2 (0.8)	2 (0.8)	/	/	/	/	7 (2.7)	/	14 (5.5)	7 (2.7)	60 (23.5)
	NOVA 4 (*n*= 37)	2 (5.4)	2 (5.4)	/	/	/	/	/	/	3 (8.1)	/	11 (29.7)
	Processed meat (*n*= 1586)	120 (7.6)	117 (7.4)	1 (0.1)	2 (0.1)	/	/	24 (1.5)	/	15 (0.9)	66 (4.2)	38 (2.4)
	NOVA 1-3 (*n*= 96)	9 (9.4)	9 (9.4)	/	/	/	/	2 (2.1)	/	2 (2.1)	12 (12.5)	/
	NOVA 4 (*n*= 1490)	111 (7.4)	108 (7.2)	1 (0.1)	2 (0.1)	/	/	22 (1.5)	/	13 (0.9)	54 (3.6)	38 (2.6)
	Animal fat products (*n*= 81)	/	/	/	/	/	/	2 (2.5)	/	/	1 (1.2)	/
	NOVA 1-3 (*n*= 77)	/	/	/	/	/	/	2 (2.6)	/	/	/	/
	NOVA 4 (*n*= 4)	/	/	/	/	/	/	/	/	/	1 (25)	/
	Meat alternatives (*n*= 165)	6 (3.6)	6 (3.6)	/	/	/	/	56 (33.9)	49 (29.7)	8 (4.8)	/	/
	NOVA 1-3 (*n*= 59)	2 (3.4)	2 (3.4)	/	/	/	/	25 (42.4)	18 (30.5)	5 (8.5)	/	/
	NOVA 4 (*n*= 106)	4 (3.8)	4 (3.8)	/	/	/	/	31 (29.2)	31 (29.2)	3 (2.8)	/	/
**SNACK**	Snack foods* (*n*= 619)	193 (31.2)	99 (16)	93 (15)	/	/	1 (0.2)	60 (9.7)	54 (8.7)	5 (0.8)	/	/
	NOVA 1-3 (*n*= 256)	56 (21.9)	34 (13.3)	22 (8.6)	/	/	/	53 (20.7)	24 (9.4)	5 (2)	/	/

		FOPNL (n (%))‡						SNRE (n (%))				
		**Any FOPNL**	**RI-Energy**	**RI-Full**	**PH**	**NS**	**MTL**	**Organic**	**Vegan**	**Non-GMO**	**Other**	**SQ**
	NOVA 4 (*n*= 363)	137 (37.7)	65 (17.9)	71 (19.6)	/	/	1 (0.3)	7 (1.9)	30 (8.3)	/	/	/
**SAUCES AND SPREADS**	Sauces (*n*= 847)	69 (8.1)	63 (7.4)	4 (0.5)	/	/	3 (0.4)	84 (9.9)	27 (3.2)	15 (1.8)	/	/
	NOVA 1-3 (*n*= 382)	27 (7.1)	23 (6)	2 (0.5)	/	/	3 (0.8)	57 (14.9)	15 (3.9)	6 (1.6)	/	/
	NOVA 4 (*n*= 465)	42 (9)	40 (8.6)	2 (0.4)	/	/	/	27 (5.8)	12 (2.6)	9 (1.9)	/	/
	Mayonnaise and dressings (*n*= 109)	23 (21.1)	22 (20.2)	1 (0.9)	/	/	/	13 (11.9)	15 (13.8)	1 (0.9)	/	/
	NOVA 1-3 (*n*= 24)	7 (29.2)	7 (29.2)	/	/	/	/	3 (12.5)	1 (4.2)	1 (4.2)	/	/
	NOVA 4 (*n*= 85)	16 (18.8)	15 (17.6)	1 (1.2)	/	/	/	10 (11.8)	14 (16.5)	/	/	/
	Sweet spreads (*n*= 101)	18 (17.8)	18 (17.8)	/	/	/	/	17 (16.8)	6 (5.9)	2 (2)	/	/
	NOVA 1-3 (*n*= 6)	/	/	/	/	/	/	5 (83.3)	2 (33.3)	/	/	/
	NOVA 4 (*n*= 95)	18 (18.9)	18 (18.9)	/	/	/	/	12 (12.6)	4 (4.2)	2 (2.1)	/	/
	Nut spreads (*n*= 68)	2 (2.9)	2 (2.9)	/	/	/	/	26 (38.2)	1 (1.5)	2 (2.9)	/	/
	NOVA 1-3 (*n*= 51)	/	/	/	/	/	/	20 (39.2)	1 (2)	2 (3.9)	/	/
	NOVA 4 (*n*= 17)	2 (11.8)	2 (11.8)	/	/	/	/	6 (35.3)	/	/	/	/
	Spreads (*n*= 171)	5 (2.9)	5 (2.9)	/	/	/	/	67 (39.2)	58 (33.9)	13 (7.6)	/	/
	NOVA 1-3 (*n*= 90)	2 (2.2)	2 (2.2)	/	/	/	/	49 (54.4)	30 (33.3)	5 (5.6)	/	/
	NOVA 4 (*n*= 81)	3 (3.7)	3 (3.7)	/	/	/	/	18 (22.2)	28 (34.6)	8 (9.9)	/	/
**SUGAR, HONEY AND RELATED PRODUCTS**	Sugar (*n*= 108)	/	/	/	/	/	/	22 (20.4)	/	2 (1.9)	/	/
	NOVA 1-3 (*n*= 108)	/	/	/	/	/	/	22 (20.4)	/	2 (1.9)	/	/
	Honey (*n*= 127)	6 (4.7)	6 (4.7)	/	/	/	/	13 (10.2)	/	4 (3.1)	4 (3.1)	/
	NOVA 1-3 (*n*= 126)	6 (4.8)	6 (4.8)	/	/	/	/	13 (10.3)	/	4 (3.2)	4 (3.2)	/
	NOVA 4 (*n*= 1)	/	/	/	/	/	/	/	/	/	/	/
	Syrup (*n*= 41)	1 (2.4)	/	1 (2.4)	/	/	/	17 (41.5)	1 (2.4)	6 (14.6)	/	/
	NOVA 1-3 (*n*= 29)	/	/	/	/	/	/	17 (58.6)	1 (3.4)	6 (20.7)	/	/
	NOVA 4 (*n*= 12)	1 (8.3)	/	1 (8.3)	/	/	/	/	/	/	/	/

RI-Energy, Reference Intakes displaying only energy value; RI Full, Nutrient specific Reference Intakes; PH, Protect Health Symbol; NS, Nutri-Score; MTL, Multiple traffic lights; Non-GMO: non-genetically modified organisms; Other EU, Other European Union and national quality schemes (displayed on Figure 1); SQ, Selected quality

* Categories with statistically significant differences in the prevalence of FOPNL between NOVA 4 and NOVA 1-3 using z-test ($p < 0.05$)

‡ Not included in the table, as they appeared only on individual products, are the Healthy Choice (three NOVA 4 Soups) and the Keyhole (one NOVA 4 Breakfast cereals, one NOVA 1-3 Dry Pasta and one NOVA 1-3 Milk alternative)
